# Bactericidal Efficacy of Ultraviolet-C Light on Virtual Reality Devices: In Vitro Assessment of Bacterial Killing

**DOI:** 10.2196/70326

**Published:** 2025-09-30

**Authors:** Scott C Roberts, Jayson Wright, Mahnoor Mustafa, Richard S Feinn, Asher Marks, Kimberly Hieftje, Pamela H Huang, Richard A Martinello, Thomas S Murray, HIPSTER Team, Eileen Blake

**Affiliations:** 1 Yale New Haven Health System, New Haven, CT, United States; 1 Department of Internal Medicine, Section of Infectious Diseases, Yale School of Medicine, 20 York Street, Hunter 527, New Haven, CT, 06510, United States, +1 203-688-2253, +1 203-688-2823; 2 Yale University, New Haven, CT, United States; 3 Quinnipiac University, Hamden, CT, United States; 4 Department of Pediatrics, Yale School of Medicine, New Haven, CT, United States; 5 Yale New Haven Health System, New Haven, CT, United States

**Keywords:** ultraviolet-C light, disinfection, virtual reality, bacteria, health care

## Abstract

**Background:**

Virtual reality (VR) headsets are increasingly used in health care settings for a variety of clinical indications, yet processes to ensure safe use between patients are not well-established. Centers vary in how these processes are performed. Most use disinfection wipes that require manual contact with VR devices for a specified dwell time to allow for sufficient pathogen killing, which may introduce manual error and device degradation over time. Ultraviolet-C light (UV-C) devices offer a no-touch, low-cost, and passive method to achieve pathogen killing without the harms of chemical contact-based disinfectants. The use of UV-C for disinfection has been studied for some medical devices but its efficacy for microbe killing on VR headsets is not well-established.

**Objective:**

This study aims to determine the bactericidal efficacy of UV-C on VR headsets through quantifying UV-C irradiance and bacterial killing of 3 commercially available UV-C devices.

**Methods:**

Three commercially available, low-cost UV-C devices were tested for UV-C energy output at multiple positions, angles, and times across the devices’ zone of disinfection. The top and lens of a VR headset, the Meta Oculus Quest 2, were artificially inoculated with high quantities of 3 different strains of bacteria (*Staphylococcus aureus, Pseudomonas aeruginosa*, and *Staphylococcus epidermidis*) and subjected to UV-C light according to each device’s manufacturer’s instructions for use. The primary outcome was the amount of bacterial killing after exposure to UV-C light.

**Results:**

All 3 UV-C devices produced a UV-C dose that ranged from 3.57 to 195.37 mJ/cm^2^, depending on proximity, angle, irradiance, and time the sensor received. At least 3-log_10_ killing of all 3 strains of bacteria was achieved for each of the tested UV-C devices; however, there was variability by organism with respect to UV-C device and VR headset location within the device, notably with the proximity of the bacteria to the bulb. *S aureus* and *P aeruginosa* were more readily killed than *S epidermidis*, with increased bacterial killing occurring with increased UV-C exposure doses. There was no experiment in which all bacteria were killed.

**Conclusions:**

UV-C dosage increased with exposure irradiance, time, proximity, and angle to the bulb for all 3 UV-C devices. Bacterial killing on the top and lens of a VR headset occurred in all 3 UV-C devices when run according to their manufacturer’s instructions for use, although full bacterial killing did not occur in any experiment. UV-C may be an effective method for microbial killing on VR equipment with low-level contamination.

## Introduction

Virtual reality (VR) devices are being increasingly used in health care settings for a variety of clinical indications in patients, as well as for education of health care personnel. VR devices are neither designed nor designated as medical products per the United States Food & Drug Administration (US FDA) [1] and typically are without manufacturer instructions for disinfection after use in health care. VR devices, usually composed of a headset and controllers, are composed of a multitude of materials, including porous material, and have complicated assembles including gaps, joints, and other surfaces that make adequate and efficient disinfection challenging. This poses a safety issue, as health care settings comprise vulnerable populations at risk of infection if pathogens are transmitted between patients and health care personnel due to inadequately disinfected devices. Outbreaks of pathogens spread by contaminated reusable medical devices have been well described [2].

We have previously demonstrated the efficacy of low-level disinfectant wipes, notably those composed of isopropyl alcohol and quaternary ammonium, in sufficiently disinfecting VR headsets with a suggested standardized operating procedure [3]. However, contact-based disinfection with wipes is susceptible to manual error, a need for prolonged dwell times of the disinfectant material, selection of the proper United States Environmental Protection Agency (US EPA)–registered disinfectant wipe according to the organisms of concern, and can be environmentally wasteful. Additionally, repeated use may damage the integrity of the devices, especially the lenses. At least 1 major VR headset manufacturer specifically recommends against isopropyl alcohol as a disinfectant for this very reason [4].

Ultraviolet-C light (UV-C), a no-touch disinfection method, is potentially an attractive alternative because it is safe, low-cost, and may not degrade the material as much as contact-based disinfectants such as bleach [5]. The standard UV-C distribution with each application avoids manual errors associated with wiping. Additionally, UV-C is known to kill organisms not routinely disinfected with most standard wipes (isopropyl alcohol and quaternary ammonium) found in health care settings, including norovirus, *Candida auris*, and *Clostridioides difficile* [6-8]. UV-C has previously been shown to achieve up to a 4-log decontamination of experimentally contaminated patient rooms with high inocula of various multidrug-resistant organisms, including methicillin-resistant *Staphylococcus aureus*, vancomycin-resistant *Enterococcus* species, carbapenem-resistant *Acinetobacter baumannii*, and *C difficile* spores, with greatest decontamination achieved with direct, as opposed to indirect, line of sight [9,10]. Newer UV-C devices have since been shown to achieve this in health care settings in as little as 5 minutes when the appropriate conditions of distance and irradiation dose are met, supporting shorter exposure times as sufficient [11]. However, many factors affect the effectiveness of UV-C disinfection, including pathogen quantity, type of dispersal (eg, drop vs spread), surface material, organic load, pathogen type, humidity, temperature, and spore formation, as well as the intensity, time, and angle of light exposure [5].

Use of VR headsets outdoors is not recommended due to potential sun damage on the lens, and direct sunlight exposure, even indoors, can result in irreversible damage in under a minute [12]. This damage is secondary to UV-A (315‐400 nanometers), UV-B (280‐315 nanometers), and UV-C (100‐280 nanometers) rays, which even at shorter wavelengths could still damage plastics and polymers found in VR headsets. To our knowledge, disinfection of VR headsets with UV-C technology has not been formally studied. If successful, this offers an alternative to disinfectant wipes as a method of safe disinfection between different users.

UV-C devices are regulated as class II medical devices according to the United States Code of Federal Regulations; however, many devices in routine use have not been approved for use by the US FDA, which requires rigorous testing to meet established regulatory standards [13]. Despite this, manufacturers of some UV-C devices market these as effective for disinfection in health care settings. In this in vitro assessment, we sought to determine the bacterial killing of 3 commercially available UV-C products, including one specifically designed and marketed for VR headset disinfection, against 3 pathogens common in health care settings artificially inoculated on VR headsets.

## Methods

### UV-C Output Measurements

**Figure 1.**
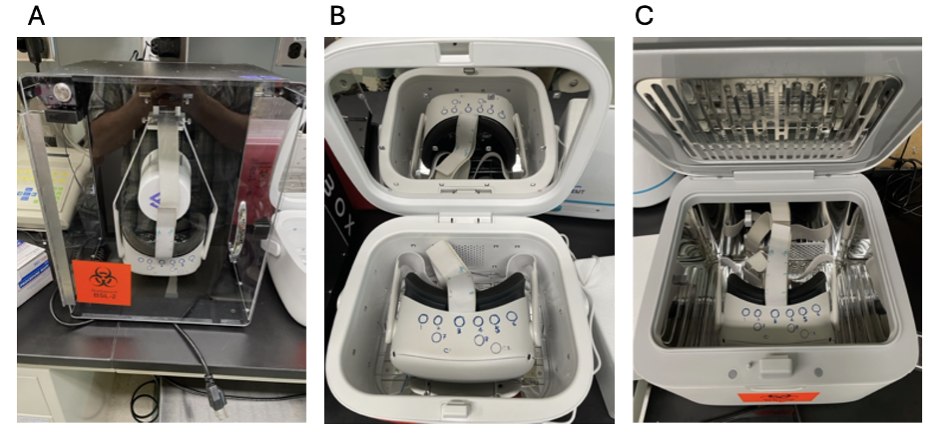
Positioning of the virtual reality (VR) headset according to ultraviolet-C light (UV-C) device. (A) In the Cleanbox, the VR headset is positioned such that the bulbs emit UV-C outward, with direct exposure to the inside strap and the lens. (B) In the LiViliti device, the bulbs emit UV-C from the top and all 4 sides with reflective material on the top and bottom. There is direct exposure to both the top and the lens. (C) In the Philips device, bulbs emit UV-C from the top of the device with reflective material on all sides. The top receives direct exposure as shown. For lens studies, the headset was rotated so that the lens faced the light source. Blue circles on the VR headset represent areas of bacterial inoculation.

Three UV-C devices were tested. The Cleanbox (Model 01‐02CX×1; Cleanbox Tech) used 265-nanometer UV-C by LED light and did not have reflective material. The LiViliti 59SUV-C LED Sanitizing Box (Model SZH40-T5; LiViliti Health Products Corp) used 260‐ to 280-nanometer UV-C by LED light and had reflective materials on the top and bottom of the device. The Philips UV-C Disinfection Box (Model 9290024876; Philips, Koninklijke Philips NV) used 254-nanometer UV-C by mercury lamp and had reflective materials on all sides of the device. The Cleanbox device was developed specifically for VR headset disinfection, while the LiViliti and Philips devices were chosen due to their low cost and advertised ability to disinfect medical devices (primarily positive pressure ventilation masks, such as continuous positive airway pressure or bilevel positive airway pressure masks). These UV-C disinfection devices are also large enough to fit the VR headset. Importantly, these latter 2 devices were not marketed for, nor reported to be tested with, VR headsets. None of these UV-C devices have been approved by the US FDA; however, they are regulated as class II medical devices. Conditions in this experiment were not designed to assess disinfection according to established regulatory standards by the US FDA, but rather this study assessed bacterial killing for 3 commonly encountered health care pathogens to determine whether UV-C devices have efficacy for VR disinfection that should be further validated to the more rigorous standards required by bodies such as the US FDA and the US EPA. While the Spaulding Classification is intended for medical devices, and VR headsets are not designated as such, they would be classified as noncritical devices that should be cleaned and disinfected with low- or intermediate-level disinfectants. The manufacturers’ instructions for use (MIFU) differed by time of exposure per device: 1 minute for the Cleanbox (Figure 1A), 3 minutes for the LiViliti (Figure 1B), and 10-20 minutes for the Philips (Figure 1C). The VR headset was placed in each UV-C device as shown in Figure 1.

This resulted in a different position of the VR device in relation to the UV-C bulbs for each device. In the Cleanbox device, the VR device was hung by a strap with the bulbs positioned between the strap and the lens. The lens received direct light but not the top of the headset. In the LiViliti device, the UV-C bulbs were positioned across the top and all 4 sides of the device, leading to direct UV-C exposure to both the top and the lens. In the Philips device, the UV-C bulbs were in the top of the device only, leading to direct UV-C exposure to the top of the device instead of the lens. The headset was rotated to provide direct lens exposure. Reflectivity also differed by device; the Cleanbox device has no reflective material to direct UV-C back toward the VR headset, while the LiViliti device has reflective material on the top and bottom and the Philips device has reflective material on all sides (Figure 1).

A UV-C sensor (X1 Optometer, Gigahertz-Optik) measured UV-C dose for each device. Initial UV-C measurements varied by time per device based on the MIFU. Additional measurements were made to compare UV-C doses across devices with 1 minute of exposure. Differences in UV-C doses based on the surface angle compared with the light source were measured by placing the UV-C sensor at different locations within the UV-C chamber of each device (center and all 4 corners) and by placing the UV-C sensor both upright or perpendicular to the top of the device. All dose measurements were performed in triplicate for each location with the average of the 3 noted in Table 1.

**Table 1.** Differences in ultraviolet-C light energy in different locations for each disinfection device.

Device (MIFU^a^ exposure time) units		Cleanbox^b^ (1 minute), mJ/cm^2^	LiViliti^b^ (3 minutes), mJ/cm^2^	Philips^b^ (10 minutes), mJ/cm^2^
Sensor placement in UV-C^c^ box and sensor position in UV-C box				
Flat facing top				
	Each corner (n=12)	3.6 (0.8)	18.6 (0.6)	215.3 (8.7)
	Center (n=3)	9.7 (0.8)	36.6 (1.0)	195.4 (4.7)
Perpendicular facing side				
	Each corner (n=12)	102.3 (33.3)	68.2 (9.8)	34.8 (9.1)
	Center (n=3)	155.8 (1.3)	53.7 (0.7)	28.4 (4.5)

a MIFU: manufacturers’ instructions for use.

b Values within parentheses reflect SD values.

c UV-C: ultraviolet-C light.

### Bacterial Killing

Bacterial inoculation and killing were performed with the same methodology as previously outlined [3]. Briefly, 3 strains of bacteria, *S aureus* (ATCC 25923), *Staphylococcus epidermidis* (ATCC 12228), and *Pseudomonas aeruginosa* (laboratory strain PAO1), were grown overnight in 3 mL of lysogeny broth (LB), serially diluted, and 10 microliters of bacteria (inoculum range 1.5×10^5^‐5.8×10^7^) were inoculated to the lens and the top of a VR headset; the Meta Quest 2 (Meta) (Figure 1). After air drying for 30 minutes, a control culture was taken to account for bacterial death during surface drying, and the VR headsets were subsequently placed in UV-C devices, as shown in Figure 1. UV-C exposure was performed according to the MIFU of each device. Following UV-C disinfection, sites of inoculation were cultured with sterile cotton swabs dipped in LB, swiped multiple times across the site of inoculation, and inoculated to LB agar plates. Plates were incubated overnight at 37 °C with bacteria colony-forming units (CFUs) counted the following day. All experiments were performed independently in triplicate for all conditions. Experiments were performed under ambient temperature and humidity to reflect real-world conditions. This was monitored in the laboratory and is typically 73‐78 °F with 20%‐35% humidity. Between experiments, the headset was sprayed with a 70% isopropyl alcohol solution to saturation and left to air dry.

### Statistical Analysis

The experimental design for the UV-C killing was a 3-factor crossed design with organism count after treatment as the outcome variable. The generalized linear model with negative binomial distribution and logit link was used to compare whether the proportion of trials with observable bacteria count after treatment differed by (1) type of UV-C disinfection box, (2) type of organism, and (3) location. All models included the natural log of bacteria count prior to treatment and used robust standard errors. The experimental design for the energy sensor was a 3-factor model with amount of energy as the outcome variable. The general linear model with normal distribution was used for comparing type of UV-C device, minutes of exposure, and location of the UV-C sensor or VR headset. For all models, pairwise comparisons were performed for UV-C devices. Analyses were performed in SPSS software (version 29; IBM Corp), and statistical significance was set at an α level of .05.

### Ethical Considerations

The laboratory experiments were exempt from Yale University Institutional Review Board evaluation, as they satisfied the Yale University 100 CH.9 Clinical Quality Improvement checklist.

## Results

### UV-C Energy Output Varies Between and Within Different Devices

**Figure 2.**
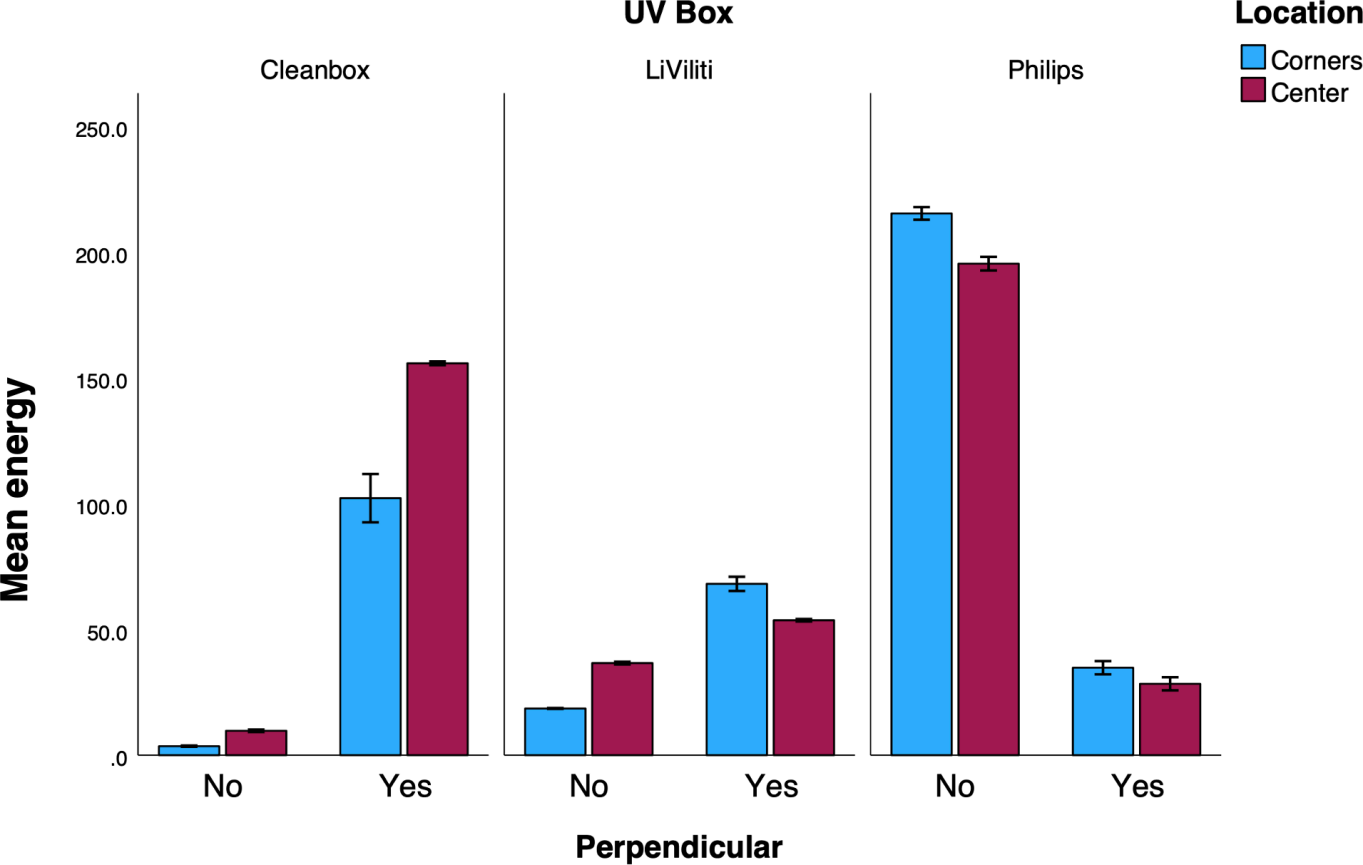
Ultraviolet-C light (UV-C) dose (mJ/cm^2^) according to UV-C device, location within each device (center [red] vs corner [blue]), and location of sensor (toward the top of the device [No perpendicular] vs toward the side of the device [Yes perpendicular]). UV: ultraviolet.

When each device was run for the time designated in the MIFU, statistically significant differences were observed correlated with the differences in the instructed run times, with the 10-minute exposure of the Philips device providing the most energy (Table 2 and Figure 2). When the sensor was placed perpendicular to the initial measurements, on the bottom of the device but facing one of the side walls, statistical differences in irradiance were again noted across all devices, likely due to the different light source locations in each device relative to the sensor angle and placement (Table 1 and Figure 2). The within-device difference was least in the LiViliti box, consistent with source locations on all 4 walls and the top of the UV-C chamber.

**Table 2.** Considerations for future use of ultraviolet-C light devices on virtual reality headsets.

Barrier to appropriate UV-C^a^ disinfection	Proposed solution	Limitations
Device fidelity over time	Measure UV-C dosage periodically	Censor accessibility, device life span
Porous material in VR^b^ headset	Place disposable barrier over porous material	Added costs, additional step, and risk of contamination during removal
Shadowing (areas not exposed to UV-C)	Complement with UV-C disinfection wipes Reposition to device to ensure direct UV-C exposure	Added costs, added time, and contact-based disinfection still needed

a UV-C: ultraviolet-C light.

b VR: virtual reality.

### Bacterial Killing by UV-C

**Figure 3.**
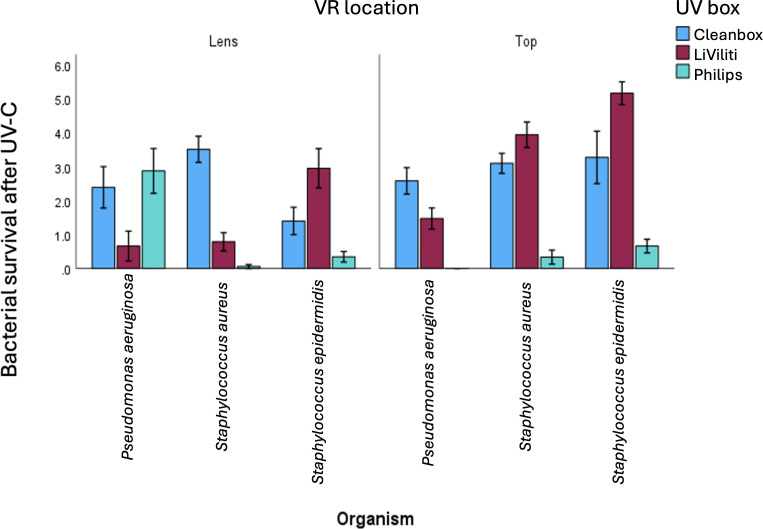
Bacterial growth by organism and UV-C device for the lens and top of each device. The y-axis represents surviving bacteria on a logarithmic scale after UV-C disinfection was performed. UV-C: ultraviolet-C light; VR: virtual reality.

There was variability of bacterial killing comparing independent replicates in mean CFU recovery according to organism and UV-C device across all tested conditions, regardless of whether the bacteria were inoculated to the top of the VR headset or the lens (Figure 3). In no experiment was there a mean of zero CFUs recovered when UV-C devices were run according to their MIFU times; there was always bacteria present. Small numbers of bacteria were recovered from some inoculated spots under all conditions tested, likely due to high inocula. However, for *P aeruginosa* and *S aureus*, a minimum of 10^3^ killing was demonstrated for all inoculated spots under all conditions tested (Figure 3; additional data not shown). There was more variation for *S epidermidis* with only the 10 minutes of exposure in the Philips device resulting in at least 10^3^ bacterial killing for all inoculated sites. For the Cleanbox device, at least 10^3^ *S epidermidis* killing was observed for all the inoculated lenses, consistent with the direct UV-C exposure, and 80% of spots inoculated on the top where there was indirect exposure to the light source (Figure 3; additional data not shown). For the LiViliti device, UV-C exposure resulted in at least 10^3^ killing for 79% of inoculated sites encompassing both the lens and the top (Figure 3).

The same VR headset used in these experiments was also used in a prior study by our group testing disinfection wipes [3]. Following completion of approximately 1860 minutes of UV-C exposure and 176 applications of chemical disinfection wipes composed of isopropyl alcohol and quaternary ammonium compounds, the device retained full functionality and had no grossly visible changes from its initial appearance.

## Discussion

### Principal Findings

In this in vitro assessment of 3 commercially available, single-use UV-C devices, we found that the recorded dosage varied depending on the location and angle of the surface, as well as MIFU time for each device. As expected, longer exposure times and closer direct surface exposure to the light source resulted in increased energy recordings. The required dosage for effective UV-C killing varies depending on the bacteria, and some degree of logarithmic reduction in bacterial counts was achieved by these devices for most organisms [9]. We attempted to adjust for potential confounders that may have led to variability in bacterial killing across organisms and devices. This included standardizing the methodology of contaminating and quantifying the bacteria on the headsets, determining the locations and positioning of bacteria on the headsets with respect to the UV-C bulb, accounting for initial inoculum counts in our statistical analysis, standardizing the dry time after inoculation prior to UV-C exposure, and including an untreated control in every experiment. Despite these measures, it is possible that other factors may have led to the observed variability in killing, including the artificially high inocula used, biofilm presence, humidity and temperature in the laboratory at the time of experimentation, and operator differences, as several individuals performed these experiments over time as would be expected in the health care setting. Finally, the required UV-C dosage required to kill bacteria may inherently differ by species. For example, predicted UV-C dosage to achieve 1-log killing of *S aureus* on surfaces is greater than that of *P aeruginosa* (66 J/m^2^ vs 55 J/m^2^) [14,15].

We found that all products reduced bacterial CFUs artificially inoculated to either the plastic top or the lens of VR headsets. In most cases, we observed at least a 10^3^ log reduction in bacterial counts after UV-C exposure, but a 10^6^ log reduction for high-level disinfection, as suggested by the US FDA and the US EPA [16], was not consistently achieved. Bacteria were recovered in all experiments, although sterilization was not the expectation. In general, the Philips device achieved higher levels of killing, especially for *S epidermidis*, and we hypothesize that this is due to the longer 10-minute MIFU time than the shorter times of the other devices. *S epidermidis* survived better than *S aureus* or *P aeruginosa* for all UV-C devices. The efficacy of UV-C killing of different organisms, including *S epidermidis,* has been shown. However, certain strains of bacteria may harbor variable susceptibilities to UV-C, and resistance after intermittent UV-C exposure has been reported [17,18]. Potential mechanisms of resistance include DNA protection and damage repair, cellular protection by pigments, and reducing photo-oxidative damage [19]. We did not test multiple strains of each organism.

### Comparison With Prior Work

These results did not achieve the level of bacterial killing previously shown for contact disinfectant wipes commonly used in health care settings, including isopropyl alcohol and quaternary ammonium wipes [3]. However, these wipes are contraindicated for use on the lens, and an alternative no-touch complimentary method is necessary. Additionally, outside of gross contamination that should be eliminated via cleaning prior to no-touch disinfection, the real-world quantity of bacteria present on VR headsets after patient use is expected to be well below the tested inocula in this study such that UV-C disinfection could remain a viable option. As an example, stethoscopes in routine use by health care workers cultured prior to disinfection have yielded ranges from 15 to several hundred bacterial CFUs, several logs lower than the quantities used for experimental contamination of the VR headsets in our study [20,21]. Studies have since shown the efficacy of UV-C disinfection on reducing bacterial CFUs on stethoscopes [22].

### Strengths and Limitations

UV-C disinfection has potential advantages compared with contact-based chemical disinfection. It results in less environmental waste, is less prone to manual error because it is passive, and it does not contain chemicals that may interact with VR-headset components. Factors that determine the efficacy of UV-C bacterial killing include the wavelength, distance from the UV-C source, and angle of contact with the surface [5]. VR headsets are shaped such that both the distance from the light source and the angle will vary, in addition to likely shadowing. This will result in different parts of the headset, such as the lens and the outside, being exposed to different levels of dosage, which will impact the efficacy of disinfection. Previously, we have shown highly effective disinfection of VR headsets for contact disinfectants commonly used in health care settings, including isopropyl alcohol and quaternary ammonium wipes [3], and were interested in whether UV-C can effectively disinfect VR headsets focusing on a high touch point surface and the lens, a surface not readily amenable to contact disinfection.

UV-C may additionally be a more attractive alternative to disinfectant wipes due to its cost-effectiveness. For example, consumer pricing of a routinely used quaternary ammonium wipe at our facility is approximately 17 cents per wipe. The lowest-priced UV-C device in our study was approximately US $150.00. Thus, cost savings could be achieved after 882 disinfection cycles, or 2.4 cycles per day if used for a year. The life span of the UV-C devices is measured in years, so with sufficient use, health care facilities that anticipate frequent device disinfection may experience significant cost savings. In our study, the most expensive device also had the lowest time per run according to the MIFU, so purchasers could consider which UV-C device to obtain depending on planned disinfection throughput.

The UV-C devices tested in this study, in contrast to disinfectant wipes used at our center, are not approved by the US FDA. However, they are regulated as class II medical devices according to the US Code of Federal Regulations; thus, the manufacturer’s instructions for use must be followed [13]. To obtain US FDA approval for use in health care, such devices need to be tested and validated for consistent bacterial killing (such as 10^6^ log killing for high-level disinfection) across a wide range of conditions using clinically relevant microorganisms beyond just bacteria. Our in vitro results showing that some bacterial killing is achieved against 3 common health care pathogens are promising, but this should be validated and expanded before such devices could be designated as medical devices by regulatory agencies, which would ensure safer use in patient care and they could be more widely recommended as a viable option.

Despite this, our experiment has found observations that may be considered should US FDA approval be pursued by UV-C device manufacturers. We suggest that users of UV-C devices for VR headset disinfection consider the following: (1) UV-C irradiance production at different locations within the device should be measured routinely to ensure that the device continues to work properly over time. While we performed this using a high-quality meter, irradiance can be less precisely confirmed using commercially available photosensitive papers specific for the measurement of UV-C [23,24]. All 3 devices continued to produce the same irradiance over the study period of approximately 1 year (data not shown). (2) Porous straps continue to require a disposable barrier for the user. This is based on the lesser efficacy of disinfection with porous than with nonporous material [3]. (3) For nonporous areas subject to shadowing, consider complementary disinfection with wipes. (4) Depending on the light source distribution within the device, it may be necessary to change the position of the VR headset in the box to ensure direct UV-C to both the high touch points outside of the VR headset and the lens, which are perpendicular surfaces. Shadowed areas, such as buttons, are less likely to receive sufficient UV-C exposure as exemplified by our sensor measurements, where placing the sensor outside of the direct line of exposure to the UV-C bulb dramatically reduced the UV-C irradiation received. In practical use, should these UV-C devices be US FDA–approved for disinfection in health care settings, patients harboring certain pathogens, such as norovirus and *C difficile*, should be restricted from VR headset use. All of the results noted are performed on clean VR devices, such that there is no gross contamination or debris noted that could impede the bactericidal efficacy of UV-C. Meticulous cleaning of all VR devices should be performed before being subjected to any attempted bactericidal method, or efficacy could be compromised in real-world settings.

We did not test spore-forming bacteria such as *C difficile* that require increased energy levels for killing but previously recommended *C difficile* infection as a contraindication for VR use in health care [3]. Importantly, only the Cleanbox is specifically marketed for UV-C disinfection of VR headsets, while the other 2 devices are not dedicated for this purpose, although none of the 3 devices are US FDA–approved as medical devices to use in health care settings. We also did not measure disinfection of hand controllers. We would expect effective UV-C disinfection with appropriate surface energy levels, although shadowing remains a concern. An additional limitation is that these in vitro findings, while promising, cannot be directly extrapolated to clinical practice as only 3 commonly encountered health care pathogens were studied, and the rigorous standards (such as testing for 10^6^ log bacterial killing as required by the US FDA and the US EPA) were not evaluated. Finally, there may have been fluctuations in the temperature and humidity in the laboratory where testing was performed, which could have impacted bacterial survival, although we attempted to control for this by doing laboratory experiments under a hood in a regulated laboratory space.

Despite these limitations, these data show that UV-C may be an effective method for bacterial killing on VR equipment with low-level contamination. We note that while we observed significant bacterial killing, in no experiment were all bacteria killed when devices were run according to their MIFU, and this work was not designed to test for formal disinfection per regulatory bodies such as the US FDA, which require additional testing before meeting such a standard. Future studies with different devices and microorganisms (we are currently testing UV-C efficacy against bacteriophages and viruses) will help clarify the optimal UV-C methodology for maximizing disinfection of VR devices should additional study to achieve this regulatory standard be pursued.

## Abbreviations

CFU: colony-forming unit
LB: lysogeny broth
MIFU: manufacturers’ instructions for use
US EPA: United States Environmental Protection Agency
US FDA: United States Food & Drug Administration
UV-C: ultraviolet-C light
VR: virtual reality
